# Probiotic and prebiotic mechanisms in IBD-associated colorectal carcinogenesis: recent advances

**DOI:** 10.3389/fnut.2025.1693875

**Published:** 2026-01-19

**Authors:** Xueru Fan, Xiaoge Wang, Yang Chao, Erping Xu

**Affiliations:** 1 Department of Gastroenterology, The First Affiliated Hospital of Henan University of Traditional Chinese Medicine, Zhengzhou, China; 2 Department of Traditional Chinese Medicine, The First Clinical Medical College, Henan University of Traditional Chinese Medicine, Zhengzhou, China; 3 Department of Traditional Chinese Medicine, Henan University of Traditional Chinese Medicine, Zhengzhou, China

**Keywords:** inflammatory bowel disease, colorectal cancer, probiotics, prebiotics, synbiotics

## Abstract

Inflammatory bowel disease (IBD), a chronic relapsing inflammatory disorder of the gastrointestinal tract, significantly increases the risk of progression to colorectal cancer (CRC). Emerging studies highlight the critical roles of gut microbial dysbiosis and sustained intestinal inflammation in driving this pathological transformation. Probiotics and prebiotics, as modulators of gut microbial ecology, have attracted considerable attention as potential interventions to restore microbial balance, regulate immune responses, and mitigate carcinogenic processes. In this review, we integrate the interplay mechanisms among inflammation, microbiota, and immunity in IBD-associated colorectal carcinogenesis (IBD-CRC), with a focus on the roles of probiotics and prebiotics in microbial remodeling, enhancement of epithelial barrier integrity, inhibition of inflammatory signaling, and activation of antitumor immunity. Furthermore, we discuss preclinical and clinical evidence supporting their efficacy in delaying or preventing IBD-CRC. The review also provides perspectives on future customized synbiotic strategies in microbiota-targeted therapy and cancer prevention.

## 1 Introduction

Inflammatory bowel disease (IBD), encompassing ulcerative colitis (UC) and Crohn’s disease (CD), is a chronic gastrointestinal disorder characterized by dysregulated immune responses and persistent inflammation. Patients with long-standing IBD face a substantially increased risk of developing colorectal cancer (CRC), especially in cases of extensive or poorly controlled disease (1). This progression exemplifies inflammation-driven carcinogenesis. Recent evidence underscores the critical role of the gut microbiota in maintaining mucosal immune homeostasis, preserving epithelial integrity, and influencing tumorigenesis. Dysbios, characterized by reduced commensal diversity and overgrowth of pathogenic bacteria, is a common feature in both IBD and CRC, contributing to barrier dysfunction and pro-inflammatory signaling (2). These interactions between inflammation, microbiota, and immunity collectively drive colorectal tumor development (3).

Probiotics, defined as viable beneficial microbes, and prebiotics, defined as selective substrates for commensal bacteria, represent emerging nutritional approaches for reshaping gut microbial ecology and function. A growing body of experimental and clinical research indicates that both probiotics and prebiotics can mitigate gut inflammation, enhance mucosal defense, and promote the production of bioactive metabolites, particularly short-chain fatty acids (SCFAs), which confer immunoregulatory and antitumor effects (4, 5).

This review summarizes current mechanistic insights into how probiotics and prebiotics influence the complex interplay among inflammation, microbiota, and immune responses in IBD-associated colorectal carcinogenesis (IBD-CRC). We highlight their roles in microbial modulation, immune regulation, and tumor suppression, providing novel perspectives for gut-targeted cancer-prevention strategies.

## 2 Microbial-immune-inflammatory interactions in IBD-CRC

**Figure 1:**
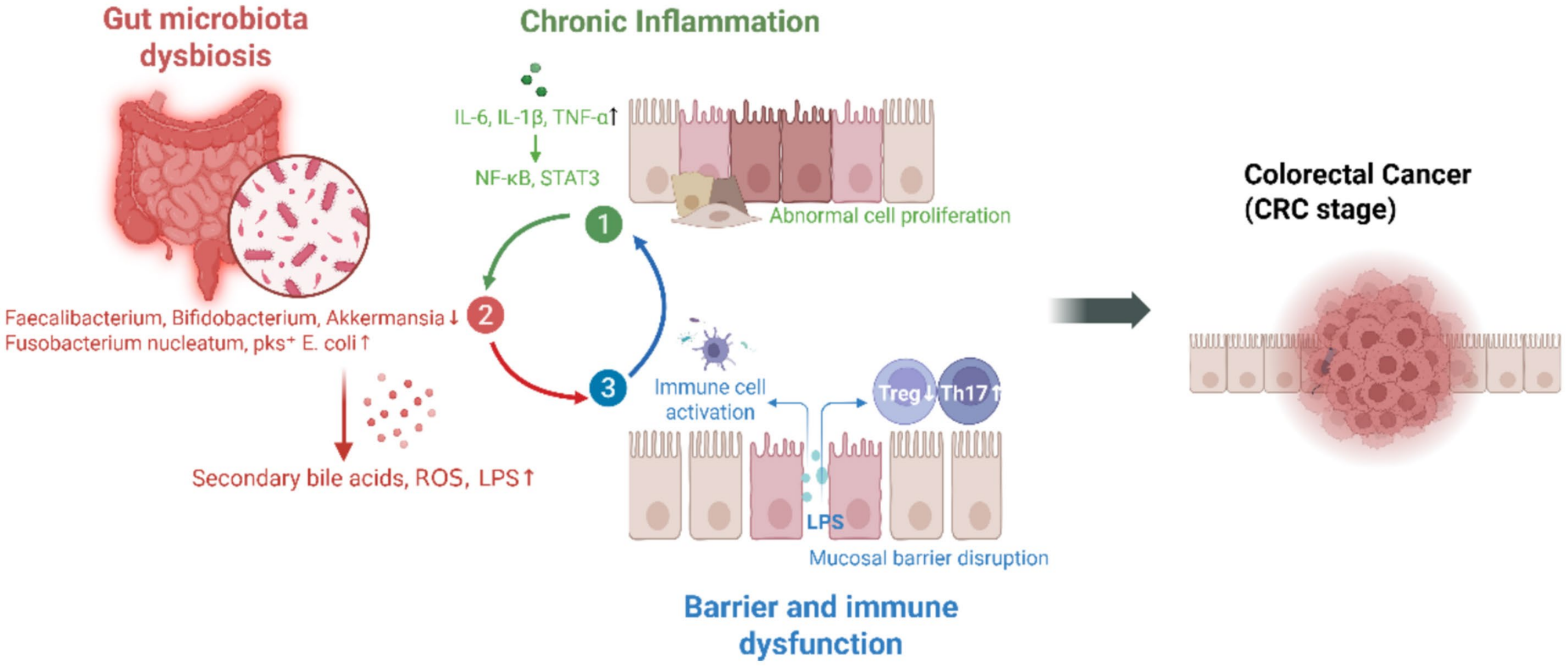
Mechanistic overview of microbiota-immune-inflammation interactions.

The pathogenesis of IBD-CRC is caused by chronic inflammation, microbial dysbiosis, and impaired barrier function, all of which collectively contribute to a tumor-promoting microenvironment (Figure 1).

### 2.1 Chronic inflammation and epithelial damage

Persistent and recurrent inflammation of the intestinal mucosa is a defining feature of IBD. Chronic inflammatory stimuli promote cycles of epithelial injury and regeneration, increasing the risk of dysplasia and malignant transformation. Key pro-inflammatory cytokines, such as TNF-*α*, IL-6, and IL-1β, activate signaling pathways including NF-κB and STAT3, which promote epithelial cell proliferation, inhibit apoptosis, and establish a microenvironment conducive to tumor development (6, 7). Importantly, these inflammatory cascades further alter the gut microbial composition and impair immune tolerance, thereby establishing a vicious cycle that drives disease progression (8).

### 2.2 Dysbiosis and microbial metabolites

IBD and CRC are both characterized by gut microbial dysbiosis, including a reduction in beneficial commensals (e.g., *Faecalibacterium prausnitzii*) and an enrichment of pro-inflammatory or genotoxic species (e.g., *Fusobacterium nucleatum*, pks^+^ *Escherichia coli*). Such dysbiosis alters microbial metabolism, resulting in the production of harmful compounds such as secondary bile acids and reactive oxygen species (ROS), which contribute to DNA damage, mutagenesis, and initiation of tumorigenesis (9). Microbial dysbiosis also exacerbates mucosal inflammation and impairs immune homeostasis, thereby amplifying carcinogenic processes (10).

### 2.3 Barrier dysfunction and immune crosstalk

In IBD, compromised intestinal barrier integrity increases epithelial permeability, facilitating the translocation of microbial components such as lipopolysaccharide (LPS) into the lamina propria. This triggers chronic immune activation and recruits innate immune cells—macrophages and neutrophils—that secrete inflammatory cytokines and ROS, exacerbating mucosal injury and tumor risk (11, 12). Moreover, impaired tolerance to commensals disturbs the balance between regulatory T cells (Tregs) and pro-inflammatory Th17 cells, leading to a dysregulated immune microenvironment that paradoxically suppresses anti-tumor immunity while promoting tumor progression (13).

## 3 Mechanisms of probiotic and prebiotic therapeutics in IBD-CRC

**Figure 2:**
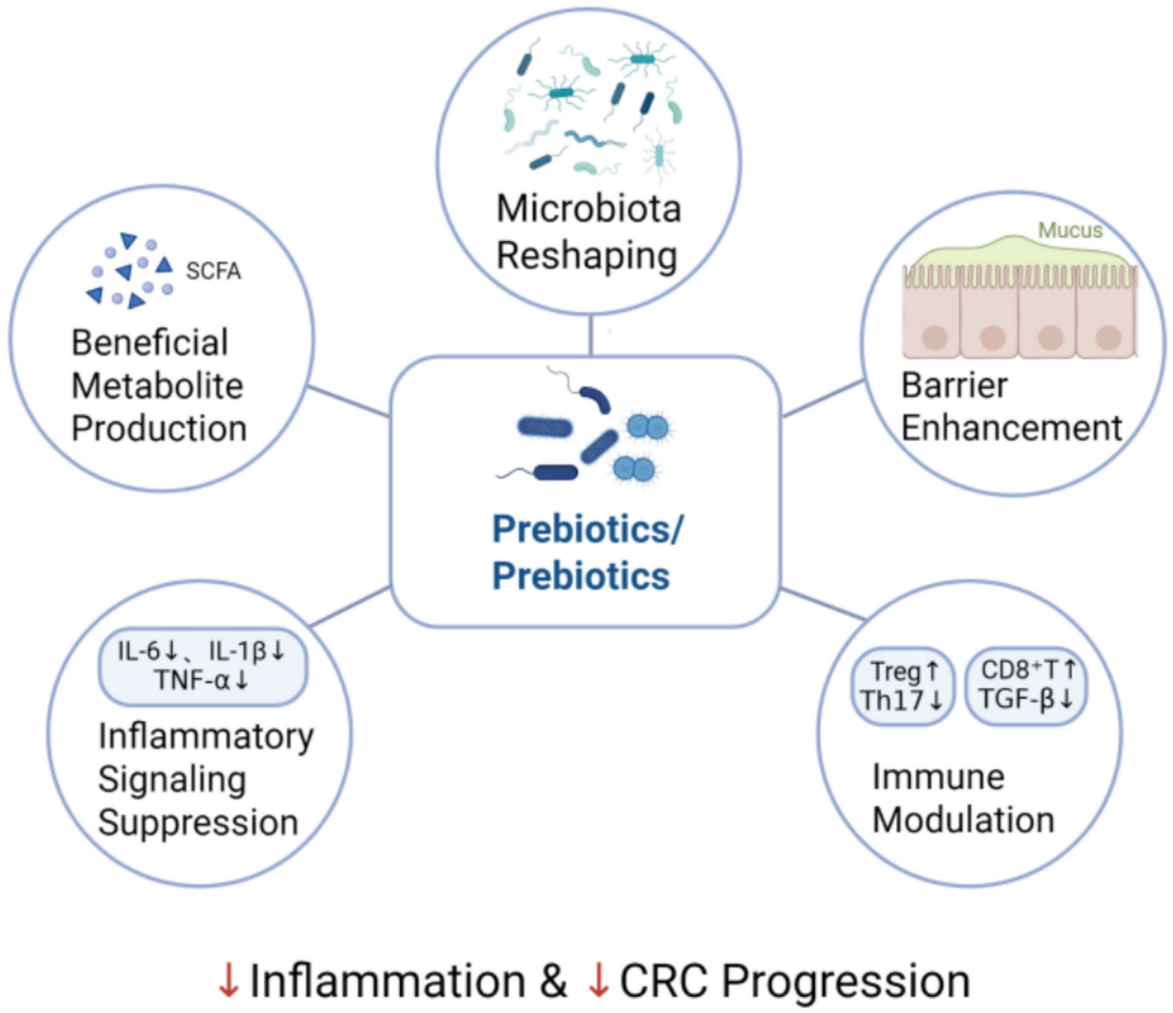
Experimental evidence pathway of probiotic/prebiotic intervention in IBD-CRC.

The therapeutic effects of probiotics and prebiotics in IBD-CRC are primarily mediated through the reshaping of gut microbiota and functional metabolite production, suppression of inflammatory signaling pathways, restoration of intestinal barrier integrity, and modulation of host immune responses (Figure 2).

### 3.1 Reshaping gut microbiota and functional metabolite production

Gut microbiota composition and diversity are fundamental to intestinal homeostasis. In IBD-CRC, dysbiosis manifests as a loss of beneficial taxa such as *Faecalibacterium prausnitzii*, *Akkermansia muciniphila*, and *Bifidobacterium* spp., accompanied by an overgrowth of pro-inflammatory and genotoxic bacteria including *Escherichia coli* (particularly pks^+^ strains) and *Fusobacterium nucleatum* (14, 15). This microbial imbalance promotes mucosal inflammation, disrupts metabolic homeostasis, and facilitates tumor development.

Probiotics restore microbial balance by introducing beneficial strains that outcompete pathogens, modulate mucosal immunity, and regulate metabolic activity. For instance, *Lactobacillus rhamnosus* GG (LGG) increases microbial *α*-diversity and decreases *Fusobacterium* abundance in colitis models (16), while *Bifidobacterium breve* M-16 V modulates immune responses by suppressing Th2-type inflammation and inducing IL-10 production (17). Prebiotics such as inulin, fructooligosaccharides (FOS), and galactooligosaccharides (GOS) selectively stimulate SCFA-producing bacteria—particularly *Faecalibacterium prausnitzii, Roseburia* spp., and *Eubacterium rectale—*thereby enriching anti-inflammatory microbial communities (18–20). Synbiotic formulations, combining specific probiotics and compatible prebiotics, further enhance these effects. For example, *Lactobacillus plantarum* combined with inulin markedly increases butyrate-producing taxa, reduces colonic inflammation, and suppresses tumor formation in preclinical studies (21).

A key consequence of this microbial remodeling is the enhanced generation of bioactive metabolites, especially SCFAs such as acetate, propionate, and butyrate. SCFAs are generated via fermentation of dietary fibers and prebiotics by commensal bacteria including *Faecalibacterium prausnitzii* and *Roseburia* spp. (22, 23). Among them, butyrate serves as a central mediator connecting microbial restoration to mucosal protection. It fuels colonocytes, strengthens epithelial integrity, and suppresses inflammatory signaling through histone deacetylase (HDAC) inhibition and activation of G-protein-coupled receptors (GPR41, GPR109A) (24). Parallel studies indicate that acetate and propionate have broader systemic actions: acetate contributes to peripheral Treg expansion and influences host metabolic pathways via GPR43/FFAR2, while propionate affects bone-marrow hematopoiesis, altering myeloid cell generation and function, and consequently attenuating systemic inflammatory responses. These mechanisms may involve GPR41/FFAR3 signaling as well as epigenetic modulation. Collectively, SCFAs act complementarily to sustain mucosal tolerance and systemic metabolic homeostasis (25, 26). Moreover, probiotics and prebiotics influence the biosynthesis of other bioactive compounds such as tryptophan-derived indoles and bile acid metabolites, which act through aryl hydrocarbon receptor (AhR) to regulate immune responses and inhibit carcinogenesis (27, 28).

### 3.2 Suppressing inflammatory signaling pathways

Chronic activation of inflammatory signaling cascades, including nuclear factor-κB (NF-κB), signal transducer and activator of transcription3 (STAT3), and mitogen-activated protein kinases (MAPKs), links persistent mucosal inflammation to neoplastic transformation in IBD-CRC (29, 30). Probiotic and prebiotic interventions mitigate these inflammatory circuits through dual mechanisms—direct microbial actions and metabolite-mediated effects, particularly those involving SCFAs.

Probiotics such as *Lactobacillus plantarum* and LGG inhibit IκB phosphorylation, thereby preventing NF-κB nuclear translocation and reducing the expression of TNF-*α* and IL-1β in colitis models (31, 32). Likewise, *Bifidobacterium infantis* downregulates STAT3 phosphorylation, suppressing epithelial hyperplasia and inflammation. Meanwhile, SCFAs, particularly butyrate generated from the fermentation of prebiotics by *Faecalibacterium prausnitzii*, *Roseburia* spp., and *Eubacterium rectale*, function as HDAC inhibitors to attenuate NF-κB and STAT3 activation, resulting in reduced IL-6 and TNF-*α* production (33–35). Prebiotics such as inulin and FOS amplify this anti-inflammatory potential by enriching SCFA-producing taxa and sustaining metabolite availability (36).

### 3.3 Repairing intestinal barrier integrity

The intestinal epithelial barrier is essential for mucosal defense, preventing luminal antigens and pathogens from translocating across the gut wall. Barrier dysfunction represents a hallmark of IBD and promotes inflammation-driven tumorigenesis (37). Probiotic and prebiotic enhance epithelial integrity via microbial and metabolite-mediated pathways that regulate tight junction proteins, mucus secretion, and epithelial renewal.

Probiotics such as LGG and *Bifidobacterium longum* strengthen epithelial cohesion by upregulating tight junction components (ZO-1, claudin-1, occludin) and activating ERK and PKC pathways that promote epithelial survival (38, 39). Concurrently, the key metabolites of prebiotics, SCFAs, support barrier function by serving as the main energy source for colonocytes and by enhancing mucin synthesis through the activation of GPR43 and GPR109A (40). Butyrate, as the most biologically active SCFA, can also inhibit the expression of myosin light-chain kinase (MLCK), thereby preventing the disassembly of tight junctions and intercellular leakage (41). Synbiotics exhibit additive effects in this process. For example, a specific synbiotic composed of FOS and eight probiotic strains has been shown to reduce intestinal permeability and restore tight junction integrity in AOM/DSS-induced colitis-associated cancer (CAC) mouse models, significantly upregulating ZO-1 (and occludin) mRNA and protein expression (42). Collectively, these interactions form a mechanistic bridge linking microbial modulation to mucosal protection.

### 3.4 Modulating host immune responses

The immune system constitutes a critical interface connecting microbial perturbations to chronic inflammation and carcinogenesis in IBD-CRC (43). Probiotic and prebiotic interventions restore immune equilibrium by jointly reshaping the gut microbiota and driving the generation of immunoregulatory metabolites. Through both direct microbial actions and metabolite-mediated mechanisms, these interventions modulate innate and adaptive immune responses to maintain intestinal homeostasis (44).

Accumulating evidence indicates that *Bifidobacterium infantis* 35,624 exerts immunoregulatory activity by enhancing IL-10 production and promoting the expansion of Foxp3^+^ regulatory T cells, accompanied by a reduction in IFN-*γ*– and IL-17–producing effector T-cell subsets in preclinical models. These immune-modulating effects appear to involve the induction of tolerogenic dendritic cells, thereby contributing to the restoration of intestinal immune homeostasis (45). Prebiotics such as inulin and GOS selectively stimulate beneficial gut microbes, especially bifidobacteria, and can further promote the enrichment of SCFA-producing taxa, including *Faecalibacterium prausnitzii* and *Roseburia* spp., through cross-feeding networks, although such increases are context-dependen (46, 47). Butyrate, the prototypical colonic SCFA, serves as a primary energy source for colonocytes and promotes colonic regulatory T-cell differentiation via inhibition of histone deacetylases and enhancement of Foxp3 expression and function (48, 49).

Beyond SCFAs, other microbial metabolites participate in immune regulation. Tryptophan catabolites (e.g., indole-3-aldehyde and indole-3-propionic acid) activate the aryl hydrocarbon receptor (AhR), promoting IL-22 secretion by innate lymphoid cells (ILC3s) and enhancing mucosal defense and repair. Secondary bile acids such as lithocholic acid (LCA) and deoxycholic acid (DCA) signal through FXR and TGR5 to suppress pro-inflammatory macrophage activation (50).

## 4 Experimental evidence from preclinical models

IBD and CRC represent distinct but interconnected stages within a pathological continuum characterized by microbial dysbiosis, barrier disruption, and chronic inflammation. The progression from IBD to CRC highlights the essential involvement of gut microbiota–immune interactions in disease development. This continuum provides a rationale for investigating probiotic and prebiotic applications as a strategic approach to intercept disease evolution, an approach supported by preclinical studies demonstrating their efficacy in ameliorating intestinal inflammation, suppressing pro-carcinogenic signaling, restoring barrier function, and modulating anti-tumor immunity (Table 1).

**Table 1:** Representative studies on probiotics and butyrate in IBD and CRC models.

Probiotic/compound used	Model type	Mechanistic target	Key findings	References
*Lactobacillus rhamnoses* GG-derived EVs	DSS-induced colitis (mice)	↑ ZO-1, ↓ TNF-α, IL-6, modulate microbiota	Restored barrier function, reduced inflammation	(38)
Sodium butyrate	TNBS-induced colitis (mice)	↓ NF-κB, ↑ GPR109A, ↑ barrier proteins	Reduced inflammation, enhanced barrier integrity	(51)
Butyrate	TNBS-induced colitis (rats)	↑ Treg, ↓ IL-17, ↓ IL-6	Induced Treg, restored immune balance	(52)
*Bifidobacterium longum* subsp.	ETEC-infected piglets	↓ *E. coli* adhesion, ↑ SCFAs	Protective effect against intestinal infections	(53)
*Akkermansia muciniphila*	AOM/DSS-induced CRC (mice)	↑ M1-like TAMs, ↑ TLR2/NLRP3	Suppressed CRC via immune activation	(54)
*Lactobacillus casei* BL23	AOM/DSS-induced CAC (mice)	↓ IL-6, ↓ tumor multiplicity	Prevented colitis-associated CRC	(55)
Butyrate	*In vitro* (colon cancer cells)	HDAC inhibition, ↑ mitochondrial metabolism	Epigenetic reprogramming and growth arrest	(56)

### 4.1 Anti-inflammatory effects of probiotics and prebiotics in IBD models

Numerous preclinical studies have demonstrated that probiotics and prebiotics possess therapeutic potential in reducing intestinal inflammation in IBD models. Commonly used animal models include dextran sulfate sodium (DSS)-induced colitis, trinitrobenzene sulfonic acid (TNBS)-induced colitis, and IL-10 knockout mice, all of which replicate critical features of human IBD pathophysiology.

Probiotic strains, including *Lactobacillus rhamnosus* GG, *Bifidobacterium breve*, and *Akkermansia muciniphila,* effectively lower the disease activity index (DAI), enhance tight junction protein expression (such as ZO-1 and occludin), and reduce pro-inflammatory cytokines such as TNF-*α* and IL-6. These benefits are linked to increased gut microbial diversity and mucus barrier repair. Prebiotics such as inulin, FOS, and resistant starch exert anti-inflammatory effects by boosting SCFA production, especially butyrate. Butyrate suppresses NF-κB activation and facilitates regulatory T-cells differentiation, thereby reducing mucosal inflammation (51, 52). Synbiotics, combining probiotics and prebiotics, provide synergistic advantages. For example, administration of *Bifidobacterium longum* with inulin reduced colitis severity and improved epithelial barrier integrity in Piglet models more effectively than either component alone (53). Collectively, these results highlight the promise of microbiota-focused therapies in reestablishing immune homeostasis, repairing barrier function, and reducing intestinal inflammation in IBD.

### 4.2 Anti-tumor effects of probiotics and prebiotics in CRC models

Preclinical studies using CRC models provided compelling evidence that probiotics and prebiotics can modulate the tumor microenvironment, inhibit carcinogenesis, and improve treatment outcomes. Commonly employed CRC models include azoxymethane/dextran sulfate sodium (AOM/DSS)-induced colitis-associated cancer (CAC), genetically modified APC^Min/+^ mice, and xenograft models.

Probiotic strains such as *Lactobacillus casei*, *Bifidobacterium lactis*, and *Akkermansia muciniphila* inhibit tumor development by lowering pro-inflammatory cytokines (IL-6 and IL-17), suppressing NF-κB and STAT3 signaling, and enhancing anti-tumor immune responses (54, 55). Notably, *Akkermansia muciniphila* restores mucosal barrier function and increase cytotoxic CD8^+^ T cell infiltration in AOM/DSS models. Prebiotics, through fermentation into SCFAs such as butyrate, exert epigenetic and immunoregulatory effects that inhibit tumors. Butyrate suppresses HDACs, enhances tumor suppressor genes (p21, Bax), and induces apoptosis in colorectal cancer cells (56). Synbiotic interventions demonstrate synergistic anti-cancer effects. For example, *Bifidobacterium longum* combined with inulin significantly reduced tumor number and volume in AOM/DSS mice, enhanced local IL-10 and IFN-*γ* expression, and restored microbiota composition (57). These findings underscore the potential of microbiota-directed interventions not only in inflammation control but also in preventing and suppressing of colorectal tumorigenesis.

Although animal studies provide strong supporting evidence, translating microbiota-based interventions to humans remains challenging due to interindividual variability and microbial diversity. Increasing attention has been directed toward synbiotic design and personalized intervention strategies.

## 5 Clinical trials and translational research

Clinical research has increasingly explored the therapeutic potential of probiotics, prebiotics, and synbiotics in IBD and CRC. Several clinical studies report beneficial effects, including reducing inflammation, promoting mucosal healing, preventing postoperative infections, and improving treatment tolerance—particularly in UC and perioperative CRC care (Table 2). However, despite these encouraging findings, translating microbiota-based interventions from experimental models into consistent clinical outcomes remains challenging. Considerable heterogeneity across trials reflects the complexity of host–microbe interactions. This underscores the need for more rigorous and stratified clinical evaluation.

**Table 2:** Clinical evidence for probiotics and synbiotics in IBD and CRC management.

Intervention	Study type /population	Clinical outcome	Key findings	References
1-Kestose (a prebiotic fructooligosaccharide)	Randomized, double-blind, placebo-controlled pilot study; Patients with mild to moderate ulcerative colitis (UC)	Clinical response and remission, symptom improvement, microbiota changes	1-kestose supplementation showed a trend toward higher clinical remission rates and improved stool form.	(58)
*Escherichia coli* Nissle 1917	Systematic review and meta-analysis (UC patients)	UC remission maintenance	Comparable to mesalazine in maintaining remission	(59)
Synbiotic Therapy	Randomized, placebo-controlled study; Patients with mild-to-moderately active ulcerative colitis (UC)	Clinical response, remission, and mucosal healing	The synbiotic group achieved significantly higher rates of clinical response and clinical remission compared to placebo. Mucosal healing was also higher.	(81)
Synbiotics (probiotic + prebiotic)	RCT (Patients with solid tumors receiving chemotherapy)	GI symptom relief, microbiota modulation	Reduced diarrhea, nausea, improved gut health	(68)
Multi-strain probiotic	Double-blind RCT (Patients undergoing colorectal cancer surgery)	Postoperative infections and barrier function	Reduced infections and enhanced tight junction protein expression	(67)

In IBD, randomized controlled trials (RCTs) investigating the probiotic strain *Escherichia coli* Nissle 1917 have shown benefits in maintaining remission in UC and preventing pouchitis, demonstrating efficacy comparable to mesalamine in several studies (58, 59). However, other RCTs have reported no significant advantage over standard therapy or placebo. Notably, negative or inconclusive outcomes occur more frequently in CD, a condition characterized by deeper transmural inflammation, distinct dysbiosis patterns, and greater ecological instability than UC (60, 61). Overall, these results highlight the complexity of clinical responses in IBD, which can be attributed to multiple layers of heterogeneity. The first level is intrinsic disease subtype differences, as UC and CD differ fundamentally in their inflammatory profiles and microbial ecology. A second layer of variability arises from strain specificity; for instance, while *Escherichia coli* Nissle 1917 demonstrates clinical efficacy, other strains such as *Lactobacillus rhamnosus* GG have failed to maintain remission in CD (62), underscoring that probiotic effects are not generalizable across species or even within genera. Methodological heterogeneity further limits cross-trial interpretation. Many studies are constrained by small sample sizes, short intervention periods, non-standardized clinical endpoints, and wide variation in probiotic dosages ranging from 10^8^ to 10^11^ CFU/day. Additionally, prebiotic interventions frequently emphasize microbiota or SCFA shifts rather than validated clinical outcomes, contributing to inconsistent interpretations (63, 64). Finally, host- and context-specific factors substantially modify therapeutic response. Baseline microbiota composition is a key determinant: individuals with low baseline butyrate production exhibit greater SCFA increases following prebiotic supplementation (65). Habitual dietary fiber intake also modulates fermentative capacity and shapes clinical benefit (66). Concomitant medications—including corticosteroids, biologics, immunomodulators, and antibiotics—further alter intestinal microbial ecology and may obscure or amplify treatment effects.

Clinical evidence in CRC remains relatively limited. Small perioperative studies suggest that probiotics may reduce postoperative infections, attenuate inflammatory responses, and mitigate chemotherapy-induced dysbiosis (67). Synbiotics have also demonstrated potential in reducing chemotherapy-related gastrointestinal adverse effects by maintaining microbial homeostasis (68). Importantly, the therapeutic context in established CRC differs markedly from that in IBD. Most CRC studies assess probiotics or synbiotics as adjuvant therapies—aimed at improving treatment tolerance, reducing gastrointestinal toxicities, and restoring microbiota balance during chemotherapy—rather than as cancer-preventive interventions. By contrast, in IBD, microbiota-directed strategies are primarily intended to prevent or delay malignant transformation by suppressing chronic inflammation, correcting dysbiosis, and reducing genotoxic microbial metabolites. These distinct therapeutic goals require different mechanistic considerations and study designs, underscoring the need for prospective trials specifically focused on the pre-carcinogenic stage of IBD-CRC.

Translational studies combining high-throughput microbiome sequencing with metabolomic profiling are increasingly revealing the basis of interindividual response heterogeneity. For example, a crossover human prebiotic trial showed that individual identity strongly determines SCFA responses, and baseline fecal SCFA levels (such as butyrate) inversely correlate with response magnitude (69). Moreover, in a metabolic syndrome cohort, baseline microbial community structure distinguished probiotic “responders” from “non-responders” (70). Additionally, in the context of immunotherapy, fecal and serum SCFAs have been proposed as predictive biomarkers of treatment efficacy (71, 72).

## 6 Future strategies and challenges in microbiota-based precision therapy

### 6.1 Synbiotic optimization: rational design of strain-substrate combinations

Synbiotics, combining probiotics and prebiotics, offer enhanced therapeutic potential by delivering beneficial microbes alongside their supportive substrates. Emerging studies show that specific strain-substrate pairs, such as *Bifidobacterium longum* and inulin, can synergistically increase SCFA production, restore epithelial barrier function, and suppress pro-inflammatory signaling. In colitis and colorectal cancer models, these combinations significantly reduce disease activity indices, inflammatory cytokine levels, and tumor burden compared to the use of probiotics or prebiotics alone (73, 74). Such synergy highlights the potential of rational synbiotic formulation as a more effective therapeutic strategy.

### 6.2 Personalization and host–microbe interactions

Interindividual variability—including microbial composition, mucosal immunity, IBD subtype, disease severity, and medication history—is a major determinant of clinical inconsistency. Certain probiotics benefit patients with identifiable microbial deficits but may exert limited effects in those with balanced microbiota (75). Multi-omics technologies enable the identification of functional microbial signatures and prediction of therapeutic responsiveness, allowing refined patient stratification and tailored synbiotic design (76, 77). Personalized strategies may include custom probiotics, targeted prebiotics, and AI-guided microbial reconstruction to maximize therapeutic efficacy while minimizing variability or adverse responses.

### 6.3 Translational barriers and regulatory challenges

Despite growing evidence for the biological relevance of microbiota-based interventions, several barriers hinder clinical translation. Response variability, driven by baseline microbiota, immune status, diet, genetics, and medication use, limits reproducibility across patient populations (78). The absence of standardized formulations, manufacturing practices, and dosing regimens further complicates evaluation. Many commercial products lack rigorous clinical validation, and their microbial viability varies widely (79). Mechanistic pathways linking microbiota modulation to host immunity and epithelial repair remain incompletely understood, underscoring the need for integrated metagenomic, metabolomic, and transcriptomic analyses (80). Additionally, live microbial therapeutics raise regulatory and ethical challenges regarding classification, safety assessment, and production standards. Well-designed clinical trials and validated biomarkers are urgently required.

### 6.4 Future perspectives: from AI to precision synbiotics

Looking ahead, the integration of AI, high-throughput sequencing, and machine learning will revolutionize microbiota-based interventions. AI can aid in predicting patient-specific responses, optimizing strain-substrate combinations, and identifying microbial signatures linked to treatment efficacy. Future directions include next-generation designer probiotics with defined immunomodulatory functions, custom prebiotics targeting microbial deficits, and AI-guided synbiotic platforms for personalized therapy. Distinguishing the preventive role of microbiota modulation in IBD-CRC from its adjunctive function in established CRC treatment will be essential for guiding future clinical trial design and precision therapy development. These innovations will facilitate the transition from empirical usage to precision microbiota medicine.

## 7 Conclusion

Probiotics, prebiotics, and synbiotics constitute a promising class of microbiota-targeted therapies capable of modulating gut microbial composition, attenuating inflammation, and disrupting colorectal carcinogenesis. This review highlights their mechanisms within the intricate interplay among inflammation, microbial communities, and host immune responses, particularly in the context of IBD-CRC. These interventions enhance microbial diversity, reinforcing epithelial barrier integrity, promote the production of beneficial metabolites such as SCFAs, and regulate immune homeostasis, thereby exerting multifaceted preventive and therapeutic effects across disease stages. Robust preclinical evidence supports their anti-inflammatory and antineoplastic properties, while emerging clinical data indicate adjunctive benefits in disease management. Despite challenges including individual heterogeneity and the need for standardized protocols, advances in multi-omics and precision medicine foster the integration of microbiome-based strategies into personalized therapeutic frameworks. Incorporating microbial-targeted approaches into dietary or nutrition-based interventions may provide new opportunities for colorectal cancer prevention. Future investigations should prioritize rigorously designed large-scale clinical trials, personalized synbiotic formulations, and identification of microbiome-derived biomarkers to optimize patient stratification and therapeutic efficacy. Collectively, microbiota-directed therapies offer a novel and integrative avenue for improving outcomes in IBD and CRC, with the potential to transform prevention, treatment, and long-term disease management.
